# Comparison of Two Botulinum Neurotoxin A Injection Patterns with or without the Medial Lower Eyelid in the Treatment of Blepharospasm

**DOI:** 10.1155/2016/5957812

**Published:** 2016-01-14

**Authors:** Hui Yang, Jing Lu, Xiujuan Zhao, Xiaohu Ding, Zhonghao Wang, Xiaoyu Cai, Yan Luo, Lin Lu

**Affiliations:** State Key Laboratory of Ophthalmology, Zhongshan Ophthalmic Center, Sun Yat-sen University, Guangzhou 510060, China

## Abstract

The aim of this study was to evaluate the efficacy of two botulinum toxin A (BoNT-A) injection patterns with or without the medial lower eyelid (MLE) in treating benign essential blepharospasm (BEB) and influencing lacrimal drainage. Two different injection patterns of BoNT-A were randomly applied to 98 eyes of 49 BEB patients: MLE Group received a full injection pattern of 5 sites and non-MLE Group received a MLE waived injection pattern of 4 sites. Tear breakup time (BUT), Schirmer I test, lagophthalmos height, and lower lid tear meniscus height (TMH) were measured and Jankovic Rating Scale (JRS) was surveyed before injection and at 1 week, 1 month, and 3 months after injection. The symptom of BEB was relieved in both groups as suggested by JRS scores at all time points after injection, and MLE Group came up with a better JRS score at 3 months. The increases of Schirmer I test value and TMH in MLE Group were higher than those in non-MLE Group at 1 week after injection. This study shows that the MLE-involved full injection pattern is a better choice for patients with BEB. It has longer-lasting effects in relieving BEB symptoms and better efficacy in reducing lacrimal drainage. Clinical Trials registration number is NCT02327728.

## 1. Introduction

Benign essential blepharospasm (BEB) is a condition of bilateral orbicularis oculi dystonia with unknown etiology, which leads to intermittent or complete involuntary eyelid closure and vision impairment [1]. Common manifestations of BEB include dry eyes, photophobia, unpleasant sensations, eyelid fluttering, and increased frequency of blinking [2]. The dysfunction might lie in the basal ganglia [3] or might be related to the impairment in corticosensory processing and a loss of inhibition of the blink reflex [4].

Botulinum neurotoxin A (BoNT-A) injection is a well-established treatment for blepharospasm and was firstly used to treat blepharospasm in 1985 [5]. The treatment efficacy lasts 3-4 months in most patients but can vary from a few weeks to more than 6 months [6]. BoNT-A injection can induce different side effects such as blurred vision, diplopia, lagophthalmos, eyelid ptosis, and increased lacrimation [7]. Injection into the pretarsal, rather than the preseptal portion of the orbicularis oculi, is more effective for treatment of BEB [8]. Also in patients with resistant blepharospasm, the pretarsal portion of the orbicularis oculi should be involved [9]. Universally used injection sites include the lateral upper and lower eyelid margins, the medial upper eyelid margin, and the lateral canthi, while additional sites differ greatly depending on patients' symptoms and the ophthalmologists' experience [8, 10–13].

However, whether the medial lower eyelid (MLE) should be involved in the treatment of patients with BEB remains unclear. Our previous study, which used a MLE-involved injection pattern, showed alleviation of dry eye symptoms via BoNT-A injection [14], while a study elsewhere, which used an injection pattern that avoided MLE, showed ineffectiveness of BoNT-A injection in treating dry eye symptoms [15]. Yet, since the injection sites of these two studies were quite different, it was impossible to make comparison and determine the effect of the MLE injection site on lacrimal drainage. Since the manifestations of BEB include dry eye, it would be of great benefit if we could improve ocular surface lubrication while treating the blepharospasm. Therefore, the aim of this study was to compare the efficacy of two BoNT-A injection patterns—with or without the MLE injection—in treating BEB and reducing lacrimal drainage.

## 2. Methods

### 2.1. Subjects

During February 2013 to December 2014 period, a total of 49 patients (13 males and 36 females; age range: 38–78 years, mean 59 years) with bilateral BEB were enrolled. The BEB symptoms should have lasted for at least 6 months prior to the baseline visit. Patients who had previously received injections of BoNT-A had to have a 24-week washout since the last injection. Exclusion criteria included blepharospasm of known etiology (caused by medication, injury, or so on), history of surgical intervention for BEB (myectomy or neurectomy), current ophthalmologic infection, and apraxia of eyelid opening associated with levator palpebrae dysfunction.

The study was approved by the Institutional Review Board of Zhongshan Ophthalmic Center, Sun Yat-Sen University (approval number 2013MEKY019), and was carried out in accordance with the tenets of the Declaration of Helsinki. Informed consent for the study was obtained from each patient at the time of enrollment.

### 2.2. Treatment

To prepare injections, 100 U of BoNT-A (Botox, Allergan, Inc., Irvine, California, USA) was diluted in 2 mL saline for a final concentration of 5 U/0.1 mL; dosage per injection was 2.5 U/site. A 32-gauge needle was used and injections were angled away from the center of the eyelid to reduce the risk of levator muscle infiltration.

**Figure 1:**
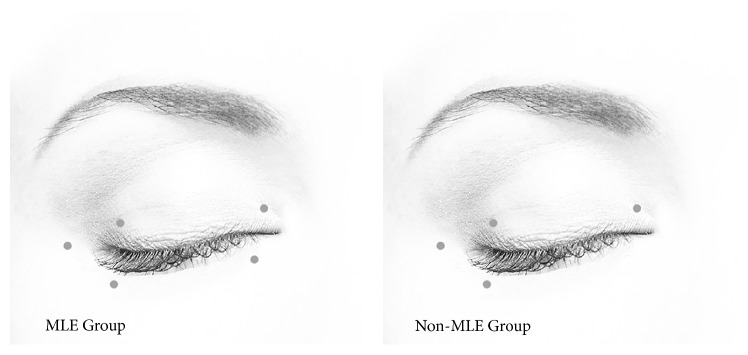
Injection sites in the MLE Group and the non-MLE Group for patients with BEB.

Each patient received 9 sites of injection (Figure 1). Right and left sides were assigned to MLE Group or non-MLE Group using a randomized digital chart. Eyes in MLE Group received a full injection pattern of 5 sites (the medial upper and lower eyelid margins, the lateral upper and lower eyelid margins, and the lateral canthi), for a total dosage of 12.5 U. Non-MLE Group eyes received a MLE waived injection pattern of 4 sites (same sites as MLE Group but excluding the MLE margin), for a total dosage of 10 U.

### 2.3. Assessments

Ocular examinations, including spasm frequency and severity, tear breakup time (BUT), Schirmer I test, lagophthalmos height, and lower lid tear meniscus height (TMH), were performed before treatment (baseline) and at 1 week, 1 month, and 3 months after injection. At each evaluation point, patients also completed a subjective questionnaire of Jankovic Rating Scale (JRS).

Tear BUT was measured as the interval between the last complete blink and the appearance of the first corneal dry spot. After one drop of fluorescein was applied to the inferior fornix, the patient was instructed to perform a complete blink and then gaze straight ahead. The mean value of three consecutive measurements was recorded. Tear BUT less than 5 sec was considered pathological.

Schirmer I test was performed with anesthesia (0.5% proxymetacaine; Alcaine, Alcon, Fort Worth, TX, USA), using a commercially available 35 mm paper strip placed on the inferior fornix between the lateral third and the middle third of the eyelid. Results were recorded in millimeters (mm) of wetting distance after 5 min; a distance less than 5 mm was considered abnormal, indicating an aqueous-deficient state.

Lower lid TMH was measured by commercial optical coherence tomography (VisanteTM OCT, version 1.0.12.1896, Carl Zeiss Meditec Inc.), from the lower eyelid-meniscus junction to the cornea-meniscus junction at the middle of the lower lid.

The JRS evaluates the severity and frequency of blepharospasm using a rating scale of symptoms from 0 to 4 [16].

### 2.4. Statistical Analysis

Data were analyzed using IBM SPSS Statistics version 20.0 (IBM Corporation, New York, USA). A level of *p* < 0.05 was considered statistically significant. Differences in Schirmer I test results between the two groups at each time point were analyzed with paired *t*-tests, while differences in tear BUT, TMH, lagophthalmos height, and JRS ratings at each time point were analyzed with Wilcoxon rank tests. Friedman and Kendall were used to make multiple comparisons between different time points for each parameter.

## 3. Results

**Figure 2:**
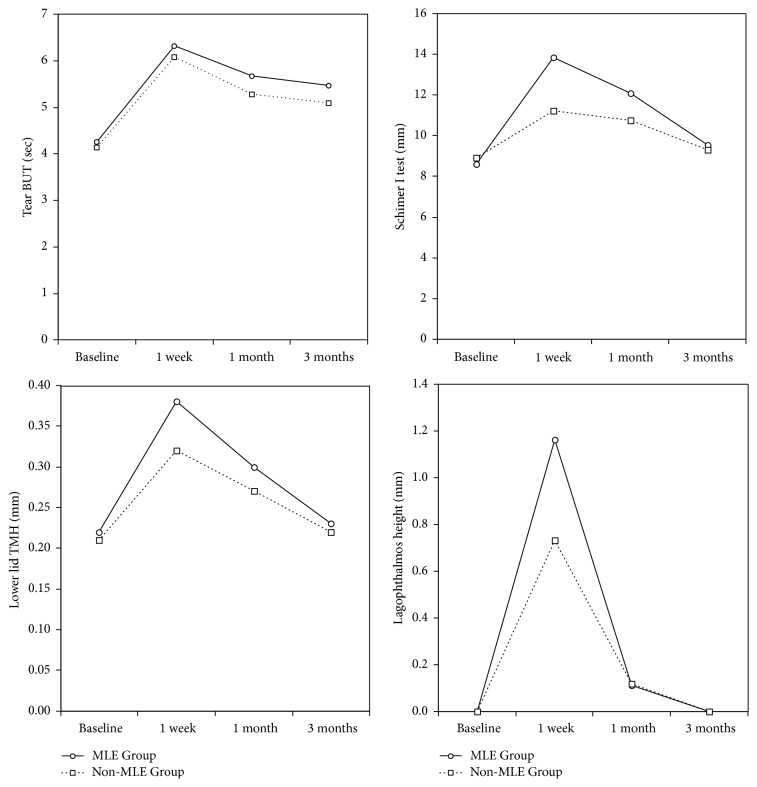
Changes in objective parameters of the MLE Group and the non-MLE Group before and after BoNT-A injection.

**Figure 3:**
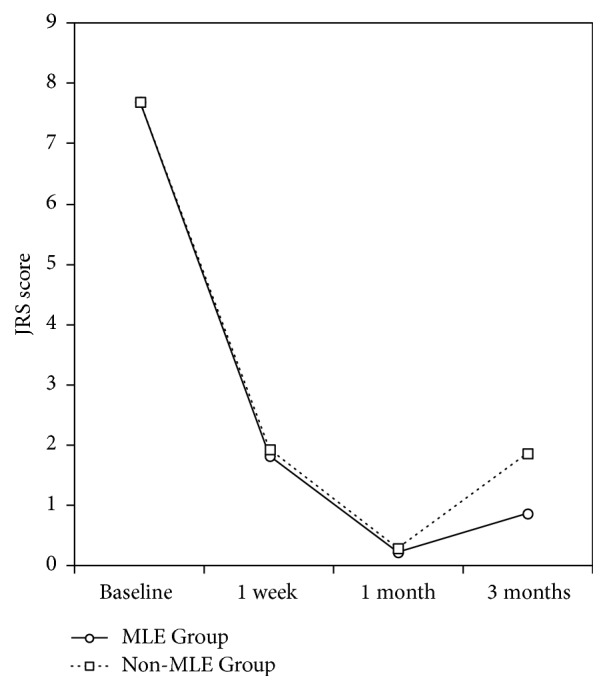
Changes in JRS score of the MLE Group and the non-MLE Group before and after BoNT-A injection.

Parameters at baseline, 1 week, 1 month, and 3 months after BoNT-A injection for each treatment group are shown in Table 1. Changes in parameters over 3 months are illustrated in Figures 2 and 3. All results in this section are given as mean and standard deviation (M ± SD).

Tear BUT, Schirmer I test value, and the lower lid TMH of the MLE Group increased significantly at Week 1 (*p* = 0.008, 0.000, 0.000, resp.) and Month 1 (*p* = 0.034, 0.014, 0.001, resp.) after injection. However, for the non-MLE Group, none of the follow-up tear BUT were statistically different from the baseline; Schirmer I test value increased at Week 1 only (*p* = 0.034); the lower lid TMH increased at Week 1 (*p* = 0.000) and Month 1 (*p* = 0.003). When comparing the two groups, the improvement in tear BUT at all time points showed no significant difference between the two groups; the increase of Schirmer I test value of the MLE Group was higher than that of the non-MLE Group at Week 1 and Month 1; the increase of the lower lid TMH of the MLE Group was more than that of the non-MLE Group at Week 1.

For both groups, the lagophthalmos height increased significantly at Week 1 (*p* = 0.000 for both groups) and returned back to baseline at Months 1 and 3. In comparison, the lagophthalmos height of the MLE Group increased more than that of the non-MLE Group at Week 1. For both groups, no ectropion, ptosis, or fluorescein staining of the cornea related to lagophthalmos was recorded, and no complaints of epiphora or diplopia were reported.

Compared to the baseline, mean posttreatment JRS scores decreased over time. There was no significant difference in JRS scores between groups at Week 1 and Month 1, but at Month 3, the MLE Group had a lower score than the non-MLE Group.

## 4. Discussion

In this study, we firstly evaluated whether the MLE site should be involved in injection of BoNT-A in treating BEB. We investigated the effects of two BoNT-A injection patterns—with or without the MLE on subjective symptoms of BEB and objective condition of the ocular surface. As suggested by JRS scores, BEB symptoms of both groups were alleviated at Week 1 and Month 1 after injection, but patients in the MLE Group had fewer complaints about blepharospasm at Month 3. This may be because an additional BoNT-A injection in the MLE chemodenervated the pretarsal orbicularis oculi, which is particularly involved in spontaneous blinking [17], more evenly. Thus, the full injection pattern had its efficacy waned at a slower rate and relieved BEB symptoms for a longer duration.

In addition, both injection patterns in this study reduced lacrimal drainage, but the full injection pattern had more noticeable effect. The lacrimal drainage may be reduced by two mechanisms, with the first being blink rate reduction. With each blink draining approximately 2 *μ*L of tears, the lacrimal drainage capacity is significantly influenced by the blink rate [18]. Therefore, less blink rate may result in less lacrimal drainage and better lubrication of the ocular surface. Second, lower eyelid laxity may play a role in lacrimal drainage reduction. Since BoNT-A injected into the MLE can paralyze the orbicularis oculi pars lacrimalis muscle, the lower eyelids and the ends of the lacrimal canals are loosened, leading to dysfunction of the lacrimal pump [19]. These explain why, in our study, eyes in the full injection pattern group had higher value of Schirmer test and TMH, while in other studies, BoNT-A injection patterns without the MLE resulted in unchanged [13] or even decreased [15] Schirmer test value.

In our study, the adverse effects of the two different BoNT-A injection patterns were also evaluated. Although some patients in both groups temporarily experienced mild lagophthalmos at 1 week after injection, no other complications such as diplopia, ptosis, epiphora, ectropion, or corneal exposure were observed. The limited adverse events in our study may be owing to the low BoNT-A dose we used, since the incidence of adverse events was dose related [7].

Although there have been several studies about the influence of BoNT-A injection on lacrimal drainage, none of them focused on the MLE injection site. In Dae Il Park's study [20], they studied the effect of BoNT-A on tear production and drainage in patients with BEB, but they did not discuss the role the MLE plays in this effect. In another study of ocular surface alterations after BoNT-A injection [21], variable injection patterns were used according to the region of contractions. Both of them emphasized the effect of BoNT-A on ocular surface condition, but they put no effort in illustrating the effect of injection sites. In Sven Sahlin's studies [19, 22], they compared two BoNT-A injection patterns with or without the upper medial lid injection in patients with dry eyes. However, the study subjects (patients with BEB) and injection sites (MLE) investigated in our study were totally different. Therefore, to the best of our knowledge, our study is the first to explore the role of the MLE in treating BEB and influencing lacrimal drainage.

In conclusion, both BoNT-A injection patterns were effective in relieving blepharospasm, but the full injection pattern involving the MLE had better efficacy at Month 3. The full injection pattern also reduced lacrimal drainage more significantly, though the influence disappeared at Month 3. Therefore, we propose that the full injection pattern, rather than the medial lower lid waived pattern, be a better choice for treating BEB.

## Figures and Tables

**Table 1:** Between-group comparisons of ocular surface parameters and subjective symptoms in the MLE Group and the non-MLE Group before and after BoNT-A injection.

		Baseline (M (SD))_0_	One week (M (SD))_1 w_	(M (SD))_1 w-0_	One month (M (SD))_1 m_	(M (SD))_1 m-0_	Three months (M (SD))_3 m_	(M (SD))_3 m-0_
Tear BUT, s	MLE	4.26 (2.83)	6.32 (3.57)	2.06 (4.33)	5.68 (3.00)	1.41 (3.61)	5.47 (3.20)	1.21 (2.65)
	Non-MLE	4.15 (2.71)	6.09 (4.20)	1.94 (4.76)	5.29 (2.69)	1.15 (3.53)	5.09 (2.21)	0.94 (2.35)
	Difference (95% CI)	0.12 (−0.31 to 0.55)		0.12 (−0.76 to 1.03)		0.26 (−0.33 to 0.86)		0.26 (−0.68 to 1.21)
	*p* value	0.608		0.747		0.409		0.807

Schirmer I test, mm	MLE	8.59 (5.09)	13.82 (6.45)	5.24 (4.92)	12.09 (5.94)	3.50 (4.32)	9.53 (4.35)	0.94 (3.27)
	Non-MLE	8.91 (4.68)	11.21 (6.00)	2.29 (4.19)	10.76 (5.27)	1.85 (4.09)	9.32 (4.05)	0.41 (2.63)
	Difference (95% CI)	−0.32 (−1.46 to 0.81)		2.94 (1.41 to 4.47)		1.65 (0.20 to 3.10)		0.53 (−0.65 to 1.71)
	*p* value	0.566		0.000^*∗*^		0.027^*∗*^		0.369

Lower lid TMH, mm	MLE	0.22 (0.13)	0.38 (0.17)	0.16 (0.17)	0.30 (0.14)	0.08 (0.12)	0.23 (0.13)	0.01 (0.07)
	Non-MLE	0.21 (0.09)	0.32 (0.14)	0.11 (0.13)	0.27 (0.12)	0.06 (0.11)	0.22 (0.09)	0.01 (0.06)
	Difference (95% CI)	0.01 (−0.02 to 0.04)		0.05 (0.01 to 0.10)		0.01 (−0.02 to 0.04)		0.00 (−0.02 to 0.02)
	*p* value	0.660		0.012^*∗*^		0.336		0.535

Lagophthalmos height, mm	MLE	0	1.16 (0.95)	1.16 (0.95)	0.11 (0.38)	0.11 (0.38)	0	0
	Non-MLE	0	0.73 (0.81)	0.73 (0.81)	0.12 (0.39)	0.12 (0.39)	0	0
	Difference (95% CI)			0.44 (0.20 to 0.67)		−0.01 (−0.06 to 0.04)		
	*p* value			0.001^*∗*^		0.655		

JRS score, points	MLE	7.70 (0.92)	1.82 (1.55)	−5.89 (1.65)	0.24 (0.83)	−7.45 (1.18)	0.88 (1.52)	−6.82 (1.65)
	Non-MLE	7.70 (0.92)	1.94 (1.48)	−5.76 (1.56)	0.30 (0.88)	−7.39 (1.20)	1.88 (1.60)	−5.82 (1.78)
	Difference (95% CI)			−0.12 (−0.29 to 0.05)		−0.06 (−0.18 to 0.06)		−1.00 (−1.35 to −0.65)
	*p* value			0.157		0.317		0.000^*∗*^

^*∗*^ *p* < 0.05.
